# Science in peril: let's fund scientific research in a sustainable and equitable way - a call for international solidarity

**DOI:** 10.17179/excli2025-8828

**Published:** 2025-11-03

**Authors:** Bachir Benarba, Atanasio Pandiella, Said Hachimi-Idrissi, Francesca Rubulotta, Eddy Lang, Abdelouahab Bellou

**Affiliations:** 1 Laboratory Research on Biological Systems and Geomatics, Mustapha Stambouli University of Mascara, Algeria; 2 Instituto de Biología Molecular y Celular del Cáncer and CIBERONC, CSIC-Universidad de Salamanca, Campus Miguel de Unamuno, Salamanca, Spain; 3 Department of Emergency Medicine, Ghent University Hospital, Ghent, Belgium; Faculty of Medicine and Health Sciences, Ghent University, Ghent, Belgium; Faculty of Medicine and Pharmacy, Vrije Universiteit Brussel, Brussels, Belgium; 4 CHIRMED Policlinico G Rodolico Catania Scuola di Anestesia, Rianimazione, Terapia Intenasiva e del Dolore, Catania, Italy; 5 iWIN ETS International women in intensive and critical care medicine network; 6 Department of Emergency Medicine, Emergency Medicine Cumming School of Medicine, University of Calgary, Alberta Health Services, Calgary, AB T2N1N4, Canada; 7 Institute of Sciences in Emergency Medicine, Department of Emergency Medicine, Guangdong Provincial People’s Hospital (Guangdong Academy of Medical Sciences), Southern Medical University, Guangzhou, 510080, China; 8 International China-Algeria Joint Laboratory on Emergency Medicine and Immunology, Guangzhou 510080, China; 9 Laboratory of Applied Molecular Biology and Immunology, Abou-Bekr Belkaïd University of Tlemcen, Tlemcen, 13000, Algeria; 10 Global Network on Emergency Medicine, Brookline, MA, USA

## ⁯⁯

In their article published in Nature (April 29, 2025), Tollefson et al. (2025[12]) asked a very important and alarming question: "Will US science survive Trump 2.0? …. What are the long-term impacts for the United States and the world?" This query calls to mind a worrying trend of political interference in research and the provision of funding as leverage against savings and national policies. In President Trump's second term, important research fields have been threatened by terminating grants, firing government scientists, planning to be reducing by billions funding to key research agencies, change international collaborations and terminating support to the World Health Organization. The decision of marginalizing work done by the scientific community is not limited to the United States; but similar challenges are registered in other countries around the world. In 2018, different amendments were changed to allow the transfer of an important part of the budget of the Hungarian Academy of Sciences to the government (Karáth, 2018[5]). Sadly, following this law research funding streams for Hungarian universities were suspended by the European Union (Koltai et al., 2024[6]). Likewise in 2021, the budget of the Brazilian research agency CAPES was reduced by 65 % from US $ 2·85 billion in 2015 to US $ 0.997 billion. Almost 90 % of the subsidy of National Fund for Scientific and Technological Development was blocked (Leandro et al., 2023[7]), which confirmed Brazilian research in a real crisis in addition to that caused by the measures taken during COVID-19. Similar trends are discernable in different countries such as India, Turkey, South Africa, and France. 

Scientific research can only prosper in an environment that respects scientists, encourages collaboration, and allows free and unconditional access to the resources needed to carry out the research institution's various projects. COVID-19 changed the landscape of clinical research (Park et al., 2021[9]). It evoked an unprecedented outpouring of activity, reflected in more than 9,700 studies registered on clinicaltrials.gov, and 443,000 publications in PubMed (accessed 28/8/2024). It showed the value of repurposing available treatments and optimizing supportive care and highlighted the power of international research collaboration by independent research networks. As far back as 1990, the Commission on Health Research and Development stated, “*Humanity's greatest health problems can be addressed most effectively through cooperative efforts by scientists around the world. Developing and developed countries and international agencies should promote and support establishment of international partnerships and steady growth in collaborative research networks as the main means for mobilizing scientific talent to attack common problems and share research strategies and results”. *Networking has a crucial role in the context of global preparedness to face emergencies such as pandemics, epidemics, wars, mass catastrophes. Authors envisage a worldwide health research system, encourage a movement to build capacity and connectivity that will foster national local regional scientific groups. Such networks should be linked together in transnational nets to address both national and global health problems” (CoHRD, 1990[1]). The first WHO Global Clinical Trials Forum in 2023 promoted an “always on, always busy” clinical research ecosystem in which “*sustained national and global clinical trial capacity during and between crises ensures … that trials can help improve health outcomes for all, all the time*” (Moorthy et al., 2024[8]). As independent research networks, and networks of those networks, we see an effective path forward to build a better and more resilient healthcare system to address future emergency needs - not only pandemics but also the burgeoning threats of the climate crisis and civil instability. Commit to an *earlier World Health Assembly recommendation *(https://apps.who.int/gb/ebwha/pdf_files/WHA58/WHA58_34-en.pdf) to *establish a minimum global target* for government support of clinical research and research capacity of 2 % of annual health care expenditures by 2030. This will subsequently have a positive and wealth-generating effect in the long term, in addition to the advances made in various scientific fields. 

The South Korean example clearly demonstrates how government investment in scientific research can contribute to scientific, technological, and economic leadership. Indeed, following the 1997 Asian financial crisis, South Korea increased its scientific research budget, making research and innovation a national priority. This contributed significantly to economic recovery and, more importantly, to its global leadership in biotechnology and semiconductors (Hemmert, 2007[3]). A recent study of OECD countries showed that public investment in basic scientific research and health services improves economic growth and long-term well-being (Parui and Prettner, 2025[10]). Likewise, Sung et al. (2025[11]) empirically demonstrated that financing R&D activities through subsidies has significant positive effects on innovation and firm productivity with measurable regional spillovers. In contrast, cutting funds allocated to scientific research will not only have negative effects on the advancement of science and its benefits for humanity, but above all will have very harmful direct effects in the long term on the economy of countries, and therefore on their international competitiveness. In a recent study published by the Institute for Macroeconomic & Policy Analysis, the long-term negative effects of cutting American federal funding for scientific research on GDP have been estimated. In fact, the study concluded that reducing public research funding would, in the long run, produce the same effect as the Great Recession: a 2.8 % reduction in US GDP, while a 50 % reduction in funding would reduce GDP by 7.6 % (González Garcia et al., 2025[2]). This reduction in GDP would, in turn, likely affect research funding. In their qualitative systems modeling analysis, Jalali and Hasgui (2025[4]) demonstrated the significant harmful effects of reducing NIH funding on the health system, including erosion of human skills, reduced life expectancy, slow therapeutic innovation, and increased health care expenditures. 

Scientists, believing that knowledge belongs to humanity and serves only its progress, must build international solidarity to ensure that scientific research is funded continuously, equitably, and transparently. 

Faced with this alarming situation, where governments are cutting funding for scientific research, it is urgent and imperative that the scientific community unite to defend science. The international scientific community, in association with learned societies, civil society, and policymakers, must advocate for:

- sustainable and unconditional funding for scientific research;
- preserving the autonomy of scientific research institutions;
- promoting policies that encourage scientific excellence, researcher mobility, and the recruitment of international talent.
- recognize the *distinctive role that acute care and emerging infectious disease research *plays in building health system resilience to the emerging threats of the 21^st^ century, and the vanguard opportunity that networks in these disciplines provide to create a truly learning health care system.

The resilience of science calls for our solidarity and collective commitment to these principles. Let us work together to ensure that science remains the foundation of human progress and hope for future generations. 

## Notes

Bachir Benarba and Abdelouahab Bellou (Institute of Sciences in Emergency Medicine, Department of Emergency Medicine, Guangdong Provincial People’s Hospital (Guangdong Academy of Medical Sciences), Southern Medical University, Guangzhou, 510080, China; E-mail: abellou402@gmail.com) contributed equally as corresponding author.

## Declaration

### Conflict of interest

The authors declare no conflict of interest.

### Artificial Intelligence (AI) - Assisted Technology 

The authors declare they have not used AI in the writing and production of the present manuscript.
